# Photosymbiosis in Late Triassic scleractinian corals from the Italian Dolomites

**DOI:** 10.7717/peerj.11062

**Published:** 2021-03-16

**Authors:** Katarzyna Frankowiak, Ewa Roniewicz, Jarosław Stolarski

**Affiliations:** Institute of Paleobiology, Polish Academy of Sciences, Warsaw, Poland

**Keywords:** Scleractinia, Carnian, Diagenesis, Symbiosis, Microstructure, Geochemistry

## Abstract

During the Carnian, oligotrophic shallow-water regions of the western Tethys were occupied by small, coral-rich patch reefs. Scleractinian corals, which already contributed to the formation of the reef structure, owed their position most probably to the symbiosis with dinoflagellate algae (zooxanthellae). Using microstructural (regularity of growth increments) and geochemical (oxygen and carbon stable isotopes) criteria of zooxanthellae symbiosis, we investigated whether this partnership was widespread among Carnian scleractinians from the Italian Dolomites (locality Alpe di Specie). Although corals from this locality are renowned from excellent mineralogical preservation (aragonite), their skeletons were rigorously tested against traces of diagenesis Irrespective of their growth forms, well preserved skeletons of corals from the Dolomites, most frequently revealed regular growth bands (low values of coefficient of variation) typical of modern zooxanthellate corals. Paradoxically, some Carnian taxa (*Thamnasteriomorpha frechi* and *Thamnasteriomorpha*sp.)**with highly integrated thamnasterioid colonies which today are formed exclusively by zooxanthellate corals, showed irregular fine-scale growth bands (coefficient of variation of 40% and 41% respectively) that could suggest their asymbiotic status. However, similar irregular skeletal banding is known also in some modern agariciids (Leptoseris fragilis) which are symbiotic with zooxanthellae. This may point to a similar ecological adaptation of Triassic taxa with thamnasterioid colonies. Contrary to occasionally ambiguous interpretation of growth banding, all examined Carnian corals exhibited lack of distinct correlation between carbon (*δ*^13^C range between 0.81‰ and 5.81‰) and oxygen (*δ*^18^O values range between −4.21‰ and −1.06‰) isotope composition of the skeleton which is consistent with similar pattern in modern zooxanthellates. It is therefore highly likely, that Carnian scleractinian corals exhibited analogous ecological adaptations as modern symbiotic corals and that coral-algal symbiosis that spread across various clades of Scleractinia preceded the reef bloom at the end of the Triassic.

## Introduction

Scleractinian corals engineer one of the most diverse ecosystems on Earth–coral reefs. Their success in oligotrophic tropical marine habitats was enabled by the emergence of symbiotic association with dinoflagellate algae (zooxanthellae) (e.g., Stanley Jr , 1981; Wood, 1999; Hallock, 2001; Kiessling, 2010). Their mutualistic relationship base on the nutrient exchange, with corals providing shelter and inorganic nutrients to their algal partners, and zooxanthellae supplying their hosts with substantial amounts of photosynthates (review in Allemand et al., 2011; Davy, Allemand & Weis, 2012). Despite the rich fossil record of scleractinian corals beginning about 245 Ma and extensive studies concerning mechanisms underlying coral-zooxanthellae symbiosis, little is known about its origin and early evolution.

The mass emergence of scleractinian corals took place in the Middle Triassic (Anisian), ca. 8–10 Ma after the end-Permian mass extinction (e.g., Flügel & Stanley Jr, 1984; Stanley Jr, 1988; Flügel & Senowbari-Daryan, 2001; Martindale, Foster & Velledits, 2019). Although already diverse in their growth form, the earliest corals only occasionally participated in the reef formation (review in Martindale, Foster & Velledits, 2019). The rapid expansion and diversification of Scleractinia occurred later, during the Late Triassic and appear to coincide with the radiation of modern dinoflagellates (MacRae, Fensome & Williams, 1996; Bucefalo Palliani & Riding, 2000; Shaked & De Vargas, 2006; LaJeunesse et al., 2018; Simpson, 2018; Mantle, Riding & Hannaford, 2020). In fact, Late Triassic coral-algae coevolution is regarded as an impetus for the success of corals as major reef builders (e.g., Stanley Jr , 1981; Stanley Jr, 1988; Stanley Jr, 2003; Flügel, 1994; Stanley Jr & Swart, 1995; Wood, 1995; Rosen et al., 2000; Payne & Van de Schootbrugge, 2007; Stanley Jr & Cairns, 1988; Kiessling, 2009; Kiessling, 2010; Martindale, Foster & Velledits, 2019). Wells (1956) hypothesized that scleractinians were initially symbiotic with algae, and asymbiotic corals appeared much later, in the Jurassic. This concept was originally supported by some molecular data showing that the oldest scleractinian lineages were associated with zooxanthellae (Barbeitos, Romano & Lasker, 2010). Consistently, fossil corals from the Middle and Upper Triassic carbonates were described as photosymbiotic (Morycowa & Szulc, 2007; Stanley Jr & Cairns, 1988; Kiessling, 2010; Stanley Jr & Helmle, 2010; Kiessling & Kocsis, 2015; Kołodziej et al., 2018) and coral-algal partnership played a key role in the Late Triassic global reef bloom—first major expansion event after the end-Permian mass extinction (Flügel & Senowbari-Daryan, 2001; Stanley Jr & Fautin, 2001; Flügel, 2002; Kiessling, 2010; Martindale, Foster & Velledits, 2019).

Although it is most commonly assumed that scleractinian corals have been symbiotic with zooxanthellae for over 230–210 myr, alternative scenarios have also been proposed. For example, Stanley Jr (1981) argued that early scleractinians originally lacked symbionts (which would be consistent with the ancestral state of the clade, see Kitahara et al., 2010; Stolarski et al., 2011), and began to be associated with algae only near the end of the Triassic. Recently, Middle to Late Jurassic (ca. 160 Mya) evolutionary divergence times among Symbiodiniaceae clades (multiple genera previously assigned to the zooxanthella genus *Symbiodinium*) were proposed by new recalibrated molecular clock based on nuclear large subunit (LSU) rDNA sequences (LaJeunesse et al., 2018). Following the LaJeunesse et al. (2018) hypothesis, the modern-day coral-algal symbioses would first emerge during the second Jurassic scleractinian radiation (not during the first Triassic one) when scleractinian communities began to build reef structures composed of lineages of modern reef corals. However, molecular analyses include only living representatives of Symbiodiniaceae and all extinct clades of the group are, by definition, excluded from consideration. It is therefore of paramount importance to provide time calibration of origin of coral-algal symbiosis based on fossil evidence.

The major difficulty in tracing symbiosis into deep time is a lack of direct evidence: no coral tissues with symbionts have been fossilized nor algal cells are incorporated into the coral skeleton. Among the variety of proposed indirect indicators of coral-algal symbiosis (e.g., Cowen, 1983; Coates & Jackson, 1987; Stanley Jr & Swart, 1995; Insalaco, 1996; Gautret, Cuif & Freiwald, 1997 Cuif et al., 1999a; Cuif, Dauphin & Gautret, 1999b; Wood, 1999; Rosen et al., 2000; Nose & Leinfelder, 1997; Helmle & Stanley Jr, 2003; Muscatine et al., 2005; Stanley Jr & Helmle, 2010; Leinfelder et al., 2006; Martindale, Bottjer & Corsetti, 2012; Frankowiak et al., 2016b; Tornabene et al., 2017), the simplest criteria are based on macromorphological differences between skeletons of modern symbiotic and asymbiotic taxa (Coates & Jackson, 1987): zooxanthellates produce highly integrated colonies with small corallites, while azooxanthellates form relatively large solitary or phaceloid skeletons. However, such simple approach appeared to be problematic because of: (i) modern exceptions like *Fungia*, *Cynarina* (both solitary and symbiotic) or *Astrangia danae* (cerioid and asymbiotic), (ii) questionable assessment of phaceloid and epithecate corals that are rare today but prevailed in the Early Mesozoic (Roniewicz & Stolarski, 1999), and (iii) disputable classification of corals with intermediate corallite size. The reliability of morphological criteria is particularly problematic when applied to fossil scleractinian taxa (e.g., Kiessling & Kocsis, 2015; Frankowiak et al., 2016a). Conversely, geochemical proxies describing variations in the isotopic composition of elements involved in algae photosynthesis (oxygen, carbon, and nitrogen), have no evident exceptions, thus can be successfully applied to ancient scleractinians (Stanley Jr & Swart, 1995; Gruszczyński et al., 1990; Muscatine et al., 2005; Frankowiak et al., 2016b; Tornabene et al., 2017). However, since diagenetic processes may obscure the original isotopic signal, the application of geochemical proxies is reasonable only with the pristine aragonite skeletons (Frankowiak et al., 2013; Tornabene et al., 2017). New opportunities to identify the former photosymbiosis are provided by microstructural observations of the fibrous part of the coral skeleton (Frankowiak et al., 2016a). This recently proposed proxy describes the differences in the regularity of the fine-scale growth increments between zooxanthellates and their asymbiotic counterparts. A considerable advantage of the microstructural criterion over geochemical characteristics is that the banding pattern can be observed even in coralla with some traces of diagenetic alteration which could affect geochemical proxies.

All Late Triassic corals examined geochemically to date were recognized as zooxanthellates (e.g., Stanley Jr & Swart, 1995; Muscatine et al., 2005; Frankowiak et al., 2016a; Frankowiak et al., 2016b; Tornabene et al., 2017), suggesting that symbiosis was common if not exclusive lifestyle among corals occupying the western Tethys. While progress continues to be made in the identification of Triassic symbiosis, the scarcity of primary preserved coral material, followed by the fact that most of the previous studies focus on fossils from one Norian site Alakir Çay Valley, Turkey (Stanley Jr & Swart, 1995; Muscatine et al., 2005; Frankowiak et al., 2016a; Frankowiak et al., 2016b; Tornabene et al., 2017) indicate that further investigations in other localities are needed. Herein, we take advantage of the well-preserved coral fauna from Alpe di Specie, Italy, in order to assess coral-algae symbiosis in the early Carnian. Although symbiosis in Carnian corals was previously addressed by Stanley Jr & Swart (1995), the application of only one symbiotic criterion to a total of five specimens points to the need for more comprehensive studies. Using high-precision analytical tools combined with various skeletal-based criteria, we investigated whether symbiosis was widespread among previously not examined Carnian scleractinian species. We have also estimated how the diagenetic alteration could influence original geochemical signatures of the examined fossil skeletons.

## Materials & Methods

### Geological setting and coral material

Fossil corals used in the present study were collected in Julian (early Carnian) deposits of the Dürrenstein/Heiligkreuz Formation in the Alpe di Specie (Seelandalpe), NE Dolomites, Italy (see Fig. 2 in Tosti et al., 2014). Despite the abundance of publications concerning the coral-bearing erratic reef boulders, their origin is still a matter of debate. They have been regarded as autochthonous remains of small shallow-water coral patch reefs (Ogilvie, 1893; Salomon, 1895) or as “Cipit-blocks”—downward displacements either from the Cassian Formation (e.g., Wendt, 1982; Sánchez-Beristain et al., 2011) or Dürrenstein/Heiligkreuz Formation (Russo et al., 1991; for a detailed discussion see Sánchez-Beristain & Reitner, 2016; Tosti et al., 2014). Leaving aside the issue of their exact origin, erratic boulders record a broad spectrum of organisms involved in the formation of Carnian reefs in this area, including calcareous sponges, corals, echinoderms, mollusks, and foraminifera, etc. The invertebrate fauna is typically characterized by excellent preservation of original skeletal mineralogy, microstructures, and geochemistry (Cuif, 1973; Montanaro-Gallitelli, Morandi & Pirani, 1974; Scherer, 1977; Laghi, Martinelli & Russo, 1984; Frisia-Bruni & Wenk, 1985; Russo et al., 1991; Nützel, Joachimski & Correa, 2010). Impermeable sediments surrounding corals provided protection from diagenesis, ensuring exceptional preservation of their skeletons (e.g., Wendt, 1977; Russo et al., 1991).

**Figure 1:**
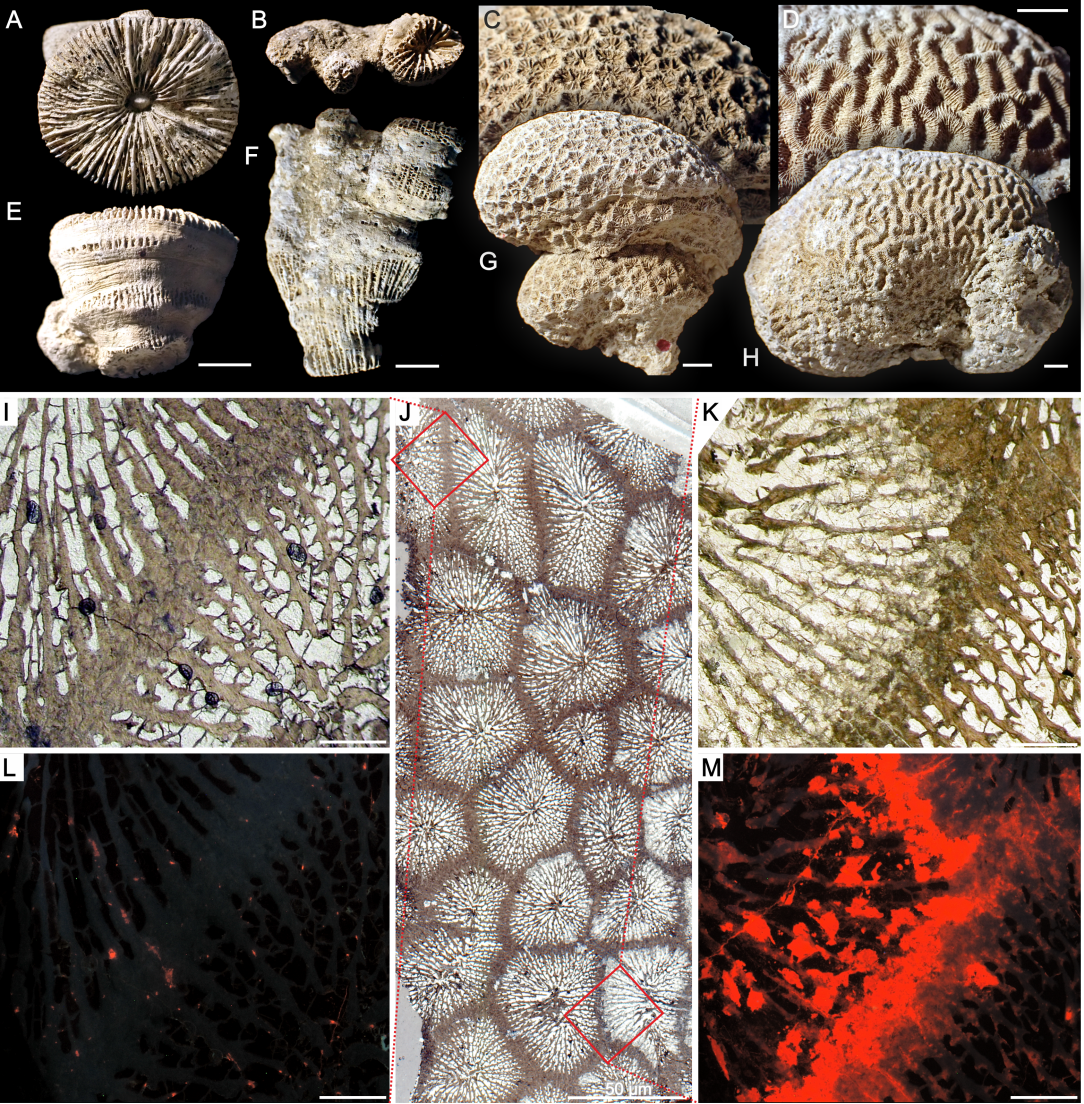
Various growth forms of scleractinian corals from Alpe di Specie and example of variable preservation within one corallum. Solitary *Craspedophyllia* sp.** (A, calicular; E, lateral views), pheceloid *Margarosmilia* sp. (B, calicular; F, lateral views), cerioid *Tropiastraea* sp. (C, detail; G, side view of the colony), meandroid *Curtoseris* sp. (D, detail; H, side view of the colony).****I–M****Transmitted light (I–K) and catholdoluminescence (L, M) photomicrographs of the cerioid colony of tropiastreiid** sp. E*.* At glance, structural characteristic of the examined colony suggests its good state of preservation (J). In close-up, parts of the colony are indeed well preserved and exhibit aragonite composition (I, L; lack of luminescence in L is indicative for aragonite), however, some regions were diagenetically altered to calcite (K, M; strong red luminescence in M is characteristic for calcite). Scale bars A–H = 5 mm, I–M = 500 μ m.

The diversified coral assemblage from the studied site shows a broad spectrum of morphologies (from solitary to complex thamnasterioid) known from modern scleractinians (Figs. 1A–1C). With few exceptions, scleractinian fauna of Alpe di Specie differs taxonomically from previously studied corals from Antalya localities (Frankowiak et al., 2016a; Frankowiak et al., 2016b) but further, in-depth taxonomic description of the coral fauna is a subject of a separate study (Roniewicz et al. in preparation). Based on observations under an optical microscope, corals with well-defined ultrastructures (Rapid Accretion Deposits - RAD and Thickening Deposits—TD; after Stolarski, 2003) were selected (Table S1). Comparative Recent material included *Leptoseris fragilis* from the Red Sea and *Montipora* sp. from Mayotte (Indian Ocean). Material is housed at the Institute of Paleobiology, Polish Academy of Sciences, Warsaw (abbreviation ZPAL).

### Analytical techniques

As a reliable interpretation of isotopic signatures of fossil samples requires using only primary aragonite material, we performed various tests against traces of diagenetic alteration in the Carnian skeletons. Following Frankowiak et al. (2013); Frankowiak et al. (2016b), the Carnian skeletons were considered as well preserved and therefore suitable for geochemical studies only when TDs met the following criteria: (a) microstructural components, especially TD, were not visually altered in comparison of those of living Scleractinia; (b) lack of Mn-induced luminescence; and (c) aragonite composition exclusively. In order to minimize possible contamination, altered TD regions, diagenetic aragonite cements on lateral faces of the skeleton, and sparry calcite from corallite infilling were excluded from geochemical sampling. Moreover, the primary area of the skeleton had to be large enough to be easily sampled (size of the drill-bit was about 350 µm). Based on these criteria, 37 specimens from the Alpe di Specie collection were selected. The microscopic and spectroscopic techniques applied for diagenetic testing are listed below.

#### Optical microscopy

Nikon Eclipse 80i transmitted light microscope fitted with a DS-5Mc cooled camera head (located at the Institute of Paleobiology, Polish Academy of Sciences) was used to assess the microstructural organization of the skeletons in thin-sections in transmitted and polarized light. Samples of Triassic corals with overall microstructural features similar to those of extant corals (though the arrangement of RAD and TD is often different from modern taxa) were considered prospective for further studies. Skeletons with different ultrastructural arrangement (e.g., composed of large sparry calcite crystals, a sign of diagenetic alteration) were excluded from further analyses.

#### Scanning Electron Microscopy (SEM)

SEM analyses were made using a Phillips XL20 scanning electron microscope at the Institute of Paleobiology, Polish Academy of Sciences to provide detailed information about crystal textures and were performed as support of transmitted-light observations. Polished sections were etched for 10 seconds in 0.1% formic acid and then rinsed with Milli-Q water and air-dried. After drying, the specimens were put on stubs with double-sided sticky tape and sputter-coated with a conductive platinum film.

#### Cathodoluminescence Microscopy (CL)

Following established procedures (Frankowiak et al., 2013; Frankowiak et al., 2016b), the hot cathode microscope HC1-LM at the Institute of Paleobiology, Polish Academy of Sciences was used to visualize cathodoluminescence of the thin-sectioned fossil coral skeletons. The following parameters were used: electron energy of 14keV and a beam current density of 0.1 µA mm-2. CL method was used to determine the spatial distribution of aragonite (original mineralogy of the skeleton) and secondary, diagenetic calcite within the skeletons. Since secondary calcite is typically characterized by high Mn^2+^ concentrations (the main activator of luminescence in carbonates) it exhibits strong orange to red luminescence (Marshall, 1988). In contrast, primary coral aragonite typically contains much lower amounts of Mn, resulting in a lack of luminescence.

#### Chemical staining with Feigl’s solution

Feigl’s chemical staining is a simple method used to identify aragonite from other carbonate minerals (e.g., calcite). The surfaces of coral skeletons were polished with an aluminum oxide suspension with 0.25 µm particle size and rinsed with distilled water. Next, the specimens were immersed in several milliliters of Feigls’s solution (Friedman, 1959) and stained for 10 minutes. The aragonite in the skeleton was stained black, whereas the calcite remained uncolored. Afterward, samples were cleaned in distilled water and dried.

#### Analysis of incremental regularity

Thickness of growth increments was measured based on SEM micrographs and transmitted light (TL) images of fossil specimens. Growth increments were measured precisely along individual fibers according to the protocol described in Frankowiak et al. (2016a). For each set of measurements taken from the individual specimen, mean value, standard deviation, coefficient of variation (CV) and confidence interval were calculated using the Past 3.02 software (Hammer, Harper & Ryan, 2001). The regularity of growth bands was expressed by the CV parameter, which describes the measure of the dispersion of values of bands thickness obtained from one skeleton. In order to check whether the results are repetitive, measurements were taken from two different specimens of the same species.

#### Oxygen and carbon stable isotopes

The samples of coral carbonate powders and corresponding infilling cement were prepared for isotopic analysis according to the method described by McCrea (1950). Next, samples (min. 20 µg) were treated with 100% orthophosphoric acid under vacuum at 70 ^∘^C in a Thermo Kiel IV Carbonate Device coupled with Finnigan Delta Plus mass spectrometer. Isotope ratios were reported in per mil (‰) delta notation relative to the Vienna Pee Dee Belemnite (VPDB) standard (defined via NBS 19). The spectrometer external error amounts ± 0.03‰  for δ^13^C and ± 0.07‰  for δ^18^O. Analyses were performed at the Institute of Geological Sciences, Polish Academy of Sciences.

## Results

**Figure 2:**
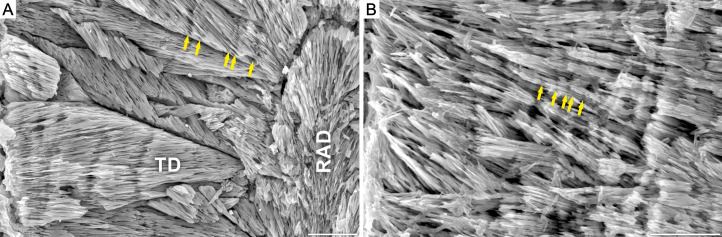
Growth increments in Carnian (Alpe di Specie) and modern scleractinian thamnasterioid corals. SEM images of fossil *Thamnasteriomorpha frechi* (A) and modern symbiotic coral *Leptoseris fragilis* (ZPAL.H.25/48) (B). Skeleton of *T. frechi* shows irregular microscale banding, that differs from regular banding pattern typical of modern zooxanthellate species (vs. generally irregular banding in asymbiotic corals). Similar irregular increments are observed in modern zooxanthellate* L. fragilis*. Note, that all living thamnasterioid are exclusively zooxanthellate, thus regular banding pattern would be expected in presented skeletons. Skeletal microstructure units: Rapid Accretion Deposits (RAD) and Thickening Deposits (TD). Scale bar 50 μ m.

**Figure 3:**
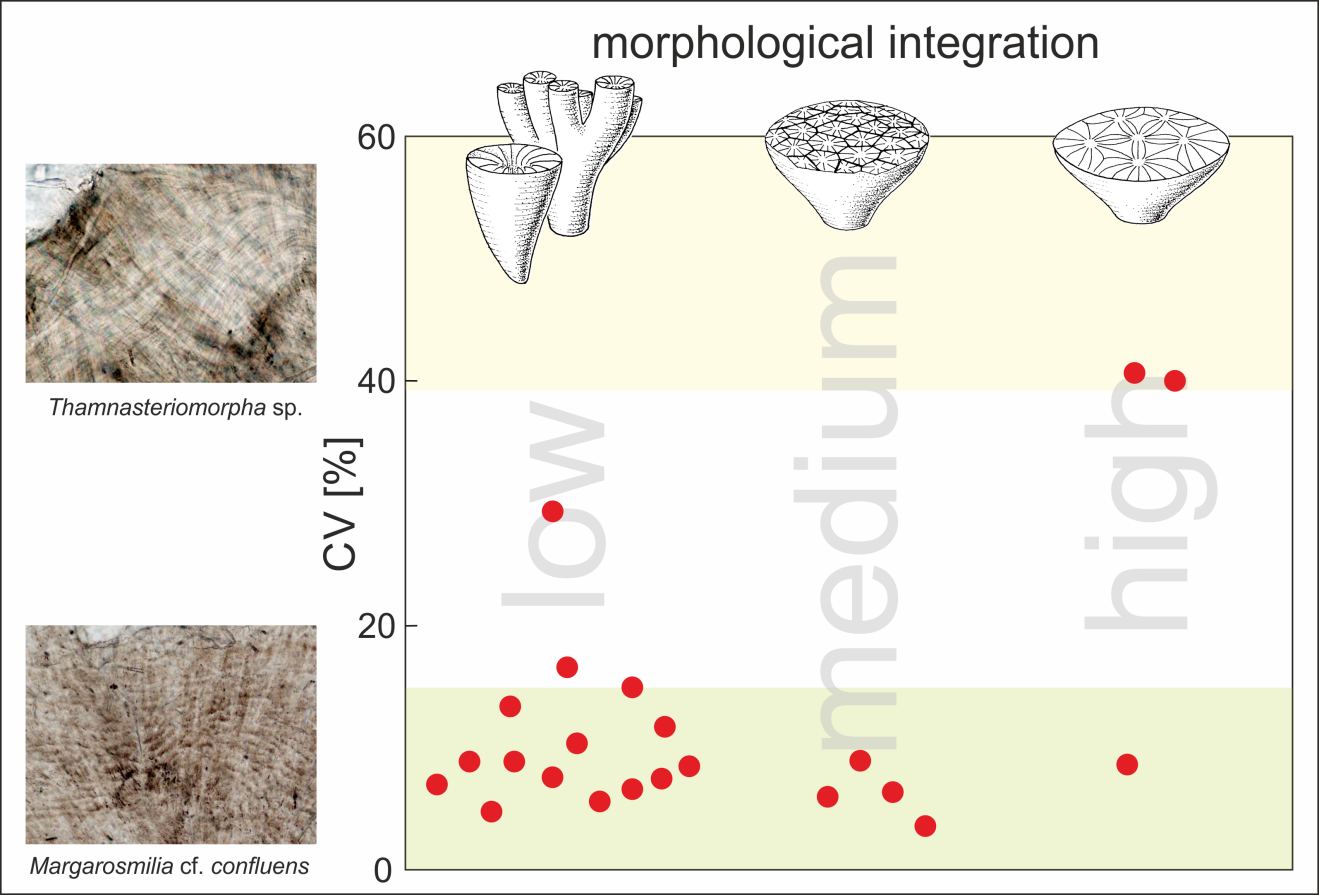
Statistical analysis of regularity of growth increments in Carnian Scleractinia from Italian Dolomites (Alpe di Specie locality). Plot shows regularity of increments (expressed as coefficient of variation [%]) in skeletons of fossil corals (red dots) with respect to the morphological form of their skeletons (low morphological integration indicates solitary and phaceloid forms; medium and high morphological integration indicate cerioid and thamnasterioid colonies, respectively). The CV values of each specimen were calculated for band thickness measured along individual fibers. Squares show transmitted light photomicrographs of the skeleton of *Thamnasteriomorpha* sp*.* with irregular bands and *Margarosmilia* cf. *confluens* with regular increments. Green shaded area corresponds to CV values characteristic for modern zooxanthellate species whereas, the yellow shaded area indicates CV values of modern azooxanthellates (based on the data presented in Frankowiak et al., 2016a). Database of all measurements is given in Table S2.

Our results show that corals from Alpe di Specie exhibit both, primary skeletal features and diagenetic alterations (Fig. 1). At the ultrastructural level, two main regions of the skeleton were distinguished: (i) the first region situated in the central regions of septa which in modern corals is recognized as composed of Rapid Accretion Deposits (RADs), and (ii) the main skeletal component recognized in modern corals as Thickening Deposits (TDs) (Fig. 2). SEM observations of Carnian specimens show that the crystal textures in the central region of septa (RADs) are typical of calcite spar (Fig. 2) and under CL exhibit strong red luminescence typical for diagenetic calcite (Fig. S2F). The susceptibility to secondary alterations RADs owe to the high amount of organic matter and nanocrystalline structure (e.g., Cuif & Dauphin, 1998; Stolarski, 2003; Stolarski & Mazur, 2005; Benzerara et al., 2011). Contrary, fibrous crystals of TDs originally consist of denser and organic-poor material that is more resistant to diagenetic processes. As a result, TDs tend to preserve their original mineralogy and microstructure in skeletons as old as Triassic (e.g., Cuif, 1977; Roniewicz, 1989). The TDs of examined Carnian skeletons locally exhibit a distinct pattern of doublets of optically dark and light bands (squares in Fig. 3). Although these bands are regarded as growth increments and thus are expected to occur commonly, their appearance in modern, as well as fossil coralla is often restricted to small areas (Fig. S1). In such optically preserved specimens, the banding pattern of alternating layers with positive and negative etching reliefs was observed under SEM (Fig. 2). Lack of luminescence indicates aragonite mineralogy of fibrous crystals (Figs. S2–S5), an observation supported by chemical staining with Feigl’s solution. Occasionally, TDs were locally altered to calcite, and these secondary areas were of few micrometers in size (Fig. S5D). The aragonite composition and microstructural arrangement argue for the exceptional preservation of the examined corals, which facilitates the study of original O and C isotopic ratios. Nevertheless, some minor altered TD regions together with diagenetic aragonite cements developed on lateral faces of the skeleton (Fig. S3J) and sparry calcite in the corallite infilling (Fig. S2–S5) were also detected.

Fine-scale skeletal banding of Carnian corals that met conditions of good preservation described above is formed by regular and continuous aragonite layers, similar to those observed in modern symbiotic corals. The regularity of these growth increments was expressed using the coefficient of variation (CV) (Table S2 and Fig. S6). Almost all examined corals, regardless of their morphological form (solitary or complex colonial), exhibit low CV between 4% and 17%, which are typical for modern zooxanthellates (green area in Fig. 3). High CV values that are placed in the range of modern asymbiotic species (yellow area in Fig. 3) were calculated only for two scleractinian specimens. Surprisingly, these corals are highly integrated *Thamnasteriomorpha* sp. (CV of 41%) and *Thamnasteriomorpha frechi* (CV of 40%). Banding measurements also show that results are repeatable, and similar CVs have been calculated for different specimens of the same species. For example, two specimens of *Remismilia* sp. have a CV of 5% and 9% respectively, and two specimens of *Margarosmilia* cf. *confluens* exhibit CV of 8% and 10%. These variations could result either from researchers’ subjective assessment, different responses to the etching or some secondary alterations that might slightly affect banding pattern –either way, these minor differences do not influence the final interpretation.

**Figure 4:**
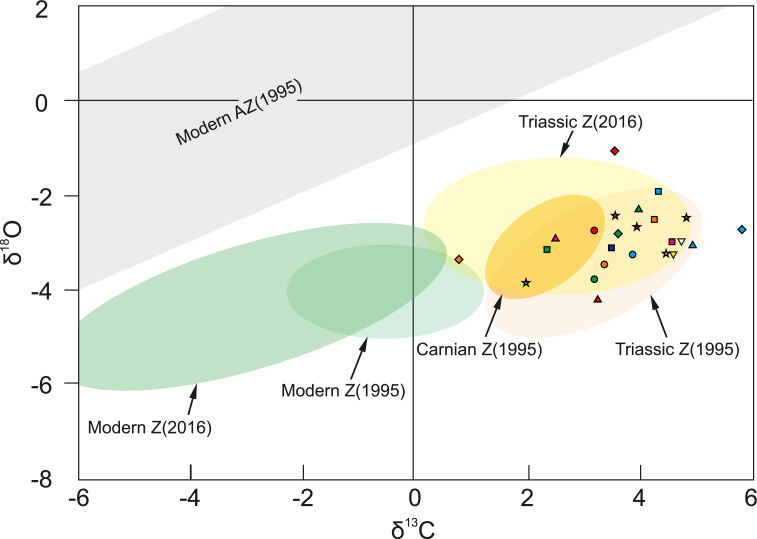
Carbon and oxygen isotopic composition of Carnian corals from the Italian Dolomites (Alpe di Specie locality). All examined fossil corals (color symbols) group in the zooxanthellate field. Pink triangle is *Thamnasteriomorpha* sp*.* and green diamond is *T. frechi*. Shaded areas show previous measurements of modern symbiotic (green) and asymbiotic (grey) scleractinians, and Triassic corals (yellow: separately Carnian and other Triassic (Norian) coral) presented by Stanley Jr & Swart (1995) and Frankowiak et al. (2016a), Frankowiak et al. (2016b); Z–zooxanthellate coral, AZ–azooxanthellate coral. Database of all measurements are given in Table S3.

Oxygen isotopic composition of corals studied herein ranges between −4.21‰  and −1.06‰ , and carbon signatures vary from 0.81‰  to 5.81‰  (Table S3). As shown in Fig. 4, examined corals tend to group together in carbon and oxygen isotope space. Possible contamination with secondary material was verified by comparison of δ ^18^O and δ ^13^C of the coral skeleton and calcite cement, and any similarities were considered as suggestive for diagenesis. However, as shown in Fig. S7 skeletons and the secondary infilling exhibit different isotopic signatures e.g., *Margarastraea klipsteini* (skeletal δ^18^O = −3.77‰  and δ^13^C = 3.19‰, calcite cement δ^18^O = −1.85‰  and δ^13^C=1.96‰), and *Tropiastraea carinata* morphotype A (skeletal δ ^18^O = −2.98‰  and δ ^13^C=4.56‰, calcite cement δ ^18^O = −5.09‰  and δ ^13^C = 0.93‰). In addition, differences in the preservation state of the skeletons are not always reflected in their isotopic composition. For example, diagenetic patterns within a single colony of the tropiastreiid sp. E range from unaltered bioaragonite to nearly completely recrystallized to calcite (Fig. 1). These regions, however, are not characterized by radically different geochemistry: the aragonite part of the skeleton exhibit δ^18^O = −3.30‰  and δ^13^C = 4.59‰, whereas secondary skeleton yield δ^18^O = −3.07‰  and δ^13^C = 4.73‰  (Fig. 4).

## Discussion

### Reconstruction of photosymbiosis

Scleractinian corals are traditionally divided into two ecophysiological groups: asymbiotic corals (typically but not exclusively deep-water) and symbiotic corals that host dinoflagellate algae (zooxanthellae) and dwell in shallow-waters (photic zone). Based on the link observed between the skeletal growth forms and coral symbiotic status, Coates & Jackson (1987) proposed a morphological criterion of symbiosis. According to these authors, zooxanthellates form highly integrated colonies with small corallites (i.e., cerioid, meandroid and thamnasterioid; <5 mm in diameter), whereas azooxanthellates form solitary or phaceloid pseudo-colonies with relatively large corallites (>5 mm in diameter). Using solely this criterion, the predominance of solitary and phaceloid growth forms within the Carnian coral assemblages (including corals from Alpe di Specie examined herein) would strongly suggest that majority of shallow-water corals from western Tethys were analogous to modern azooxanthellates (for taxonomic and growth form overview see: Roniewicz & Morycowa, 1989; Riedel, 1990; Turnšek & Senowbari-Daryan, 1994; Roniewicz & Michalik, 2002; Schäfer, 1979; Flügel, 1981; Wurm, 1982 Stanley Jr & Senowbari-Daryan, 1986; Stanton & Flügel, 1989; Stanton & Flügel, 1987; Bernecker, Weidlich & Flügel, 1999; Flügel & Senowbari-Daryan, 2001; Flügel, 2002; Bernecker, 2005; Zonneveld et al., 2007; Roniewicz, 2011; Martindale, Bottjer & Corsetti, 2012; Peybernes, Chablais & Martini, 2015; Stanley Jr & Onoue, 2015). Such conclusions would match reconstructions proposed for the entire Triassic and Jurassic scleractinian fauna (Kiessling & Kocsis, 2015). However, all sufficiently well preserved coral from Alpe di Specie locality (including taxa with solitary and phaceloid growth forms) exhibited a regular pattern of micro-scale growth increments and narrow ranges of isotopic compositions, typical of modern zooxanthellates. These observations support the hypothesis that coral-algal symbiosis was established prior to the Norian-Rhaetian massive reef bloom and that symbiotic were taxa representing a much wider spectrum of growth forms in comparison to modern coral fauna (Veron, 1995; Kiessling et al., 2009; Kiessling & Kocsis, 2015; Frankowiak et al., 2016b). Our results thus extend Stanton’s (2006) conclusion that the skeletal macromorphology, although helpful in inferring the presence of photosymbiosis in ancient coral assemblages regarded as a whole, can be misleading for particular species (which in the Triassic were dominated by taxa of simple morphological forms).

The regularity of growth bands within skeletons of corals from Alpe di Specie strongly suggests light-controlled responses. As the regular secretion of skeletal carbonate in modern corals is linked to the diurnal, cyclical activity of photosynthesizing algae (e.g., Moya, 2006; review in Allemand et al., 2011; Levy et al., 2011; Sorek et al., 2013; Sorek et al., 2014; Inoue et al., 2018) we assume that the banding pattern of studied corals results analogously from the symbiosis. To optimize photosynthetic energy acquisition that allows coral holobiont (coral host with endosymbiots) to thrive in oligotrophic tropical settings, corals had to develop various strategies ensuring efficient collecting and processing of light. It was observed that modern corals exhibit different ecomorphotypes, some of which seem to increase light-capturing abilities of endosymbionts e.g., platy morphology is a common feature for zooxanthellate corals living in deeper, poorly-lit waters (Insalaco, 1996; Rosen et al., 2000; Martindale, Bottjer & Corsetti, 2012). However, such an approach can be misleading in case of solitary and phaceloid taxa. Enríquez et al. (2017) showed that solitary corals also exhibit high light-scattering abilities, and even corals with less efficient optical properties with phaceloid growth forms, may develop robust holobionts. For example, phaceloid *Retiophyllia* corals formed very large colonies (up to 4 m) that contributed to the formation of the Early Norian patch reef formation (Martindale et al., 2013).

Many studies show that photosynthetic activity affects coral carbon isotopic pool (e.g., Swart, 1983; McConnaughey, 1989; Allison, 1996; McConnaughey et al., 1997; Grottoli & Wellington, 1999; Reynaud et al., 2001, see review in Grottoli, 2000; Linsley et al., 2019), resulting in higher values of δ^13^C in the skeleton of zooxanthellate corals in comparison to the azooxanthellate forms inhabiting similar environment (e.g., Stanley Jr & Swart, 1995; Frankowiak et al., 2016b). In contrast, oxygen isotopic signatures of the skeletons are not directly influenced by endosymbiotic algae (e.g., Swart, 1983; Juillet-Leclerc, 2019), however, variations in δ^18^O between different taxa are considered to originate from “vital effects”–biologically controlled modifications of skeletal geochemistry (e.g., Urey et al., 1951; McConnaughey, 1989; review in Schoepf et al., 2014; Devriendt, Watkins & McGregor, 2017). When combined, the δ^18^O and δ^13^C parameters differ significantly between symbiotic and asymbiotic scleractinians (Fig. 4; Swart, 1983; Stanley Jr & Swart, 1995; Cohen & McConnaughey, 2003; Adkins et al., 2003; Frankowiak et al., 2016b; Prada et al., 2019). The oxygen and carbon isotopic signatures from Alpe di Specie corals fall within the range of values measured for Late Triassic scleractinians previously recognized as symbiotic (yellow ellipse in Fig. 4; Stanley Jr & Swart, 1995; Frankowiak et al., 2016b) and do not show a clear correlation (*r*^2^ = 0.1 for linear regression) which is consistent with similar observation for modern zooxanthellate and in contrast to azooxanthellate corals (Stanley Jr & Swart, 1995; Prada et al., 2019). These observations corroborate with photosymbiotic status of all studied herein corals.

### Exceptions

Our study revealed also an irregular character of growth increments in highly integrated colonies of *Thamnasteriomorpha* sp. and *Thamnasteriomorpha frechi*. These microstructural traits may suggest their asymbiotic status. However, the oxygen and carbon isotope signatures are like those of modern symbiotic coral skeletons (Swart, 1983; McConnaughey, 1989; McConnaughey et al., 1997; Grottoli & Wellington, 1999; Devriendt, Watkins & McGregor, 2017). The possible modern analog of such microstructural and isotope data discrepancy is symbiotic deep-water coral *Leptoseris fragilis*. Similar to Triassic specimens, *L. fragilis* forms thamnasterioid colonies with irregular banding patterns (Frankowiak et al., 2016a; Fig. 2). Moreover, both *L. fragilis* and two examined species of *Thamnasteriomorpha* have septal faces covered by menianes—a ledge-like structures, which in modern *L. fragilis* serve to hold gastric ducts responsible for suspension-feeding (Schlichter, 1991). Structural similarities between these corals may suggest that *Thamnasteriomorpha* was also symbiotic but adopted to suspension-feeding trophic strategy. Similar conclusions were drawn also about the symbiotic mode of life of Jurassic microsolenid corals (Leinfelder, 1994; Insalaco, 1996; Morycowa & Roniewicz, 1995). Nonetheless, unique gastral anatomy and mode of feeding were recognized only in modern *L. fragilis* (Schlichter, 1991) and not confirmed/recognized in other modern taxa that develop menianes (e.g., *Dactylotrochus*, see Kitahara et al., 2012). Consequently, suspension-feeding strategy of all menianae-bearing Carnian species remains speculative.

### Triassic history of coral-algae symbiosis

The earliest scleractinian corals appeared in the Anisian, about 8–10 Ma after extinction at the end of the Permian (e.g., Flügel, 2002; Martindale, Foster & Velledits, 2019). Although already diverse and complex in skeletal morphologies, these corals played minor role in reef and non-reef marine ecosystems (Kolosváry, 1958; Scholz, 1972; Mostler, 1976; Deng & Kong, 1984; Qi, 1984; Martin & Braga, 1987; Morycowa, 1988; Morycowa, 1990; Richter, 1999; Emmerich et al., 2005; Deng, 2006; Morycowa & Szulc, 2007; Morycowa & Szulc, 2010). The Middle Anisian to Carnian reefs were dominated by sponges, while reef-dwelling corals rarely participated in the framework construction (review in Martindale, Foster & Velledits, 2019). Scleractinians emerged as a structure-building component of reefs later at the Carnian-Norian transition. This interval marks the beginning of Carnian-Rhaetian taxonomic turnover among coral species, with the most intense reef period, that of the Norian “global reef-bloom”(e.g., Stanley Jr & Swart, 1995; Flügel & Senowbari-Daryan, 2001; Flügel, 2002; Stanley Jr, 2003; Stanley Jr & Van de Schootbrugge, 2009; Kiessling, 2010; Martindale, Foster & Velledits, 2019. A massive expansion in the number of reefs, since then built primarily by corals, was followed by an increase in their latitudinal range that slightly exceeded limits of modern tropical coral reefs (Kiessling, 2010). Late Triassic emergence of the coral reefs was extensively discussed and explained by the appearance of symbiosis with zooxanthellae (e.g., Stanley Jr , 1981; Flügel, 2002; Stanley Jr, 2003; Stanley Jr & Van de Schootbrugge, 2009; Kiessling, 2010.

The warm tropical climate conditions of the Late Triassic favored the extensive development of carbonate platforms and reefs at the western margin of the Tethys. The shallow-water seas were characterized by normal marine salinities and sea-surface temperatures (SST) of 27–32 ^∘^C (Nützel, Joachimski & Correa, 2010). Although data concerning nutrient dynamics and its exact availability in the Triassic seas are still scarce, the former status of the Late Triassic Ocean has been inferred as oligotrophic (Riedel, 1991; Nützel, Joachimski & Correa, 2010; Martindale et al., 2015). Consistently, recent studies of nitrogen isotopic composition of the intraskeletal organic matrix show that Late Triassic corals from NW Tethys inhabited nutrient-poor waters, similar to modern Bermuda (Frankowiak et al., 2016b; Tornabene et al., 2017). Reconstructed environmental conditions roughly correspond to present-day shallow-water tropical reefs, suggesting that like in modern reef corals, symbiosis was the most profitable adaptation for the early scleractinians. By supporting the host’s energy demands for metabolism, growth, reproduction, and calcification, zooxanthellae would facilitate corals diversification and expansion in Triassic oceans (e.g., Stanley Jr, 1981; Stanley Jr, 1988; Cowen, 1988; Stanley Jr & Swart, 1995; Stanley Jr & Helmle, 2010; Kiessling, 2010). Furthermore, such enhancement would constitute a major advantage over other reef constructors (coralline sponges, calcareous algae or “*Tubiphytes*”; Flügel, 2002) in the competition for benthic substrate, and thus promoting the evolution of scleractinians into prominent builders (e.g., Chadwick & Morrow, 2011). Both, ecological and evolutionary benefits of the early photosymbiosis are indisputable, however, it remains to be clarified what stimuli triggered corals and algae to establish such close relationship. Was the appearance and spread of this partnership governed by environmental factors or was it self-organization mechanism? Kiessling (2010) postulated that photosymbiosis was related to long-term climate cooling and the decline of CO_2_ levels, that forced zooxanthellae into coral polyp tissue. On the other hand, many studies show that the coral host, rather than the algae dictate the symbiotic relationship (review in Davy, Allemand & Weis, 2012).

The patch reefs from Alpe di Specie constitute one of the earliest examples of skeletal framework biotically similar to modern tropical reefs composed mainly of corals and coralline algae (Tosti et al., 2014). The coral fauna exhibit a diverse array of growth forms known from living reef-building corals: highly integrated colonies (cerioid, meandroid, and thamnasterioid) as well as solitary and phaceloid forms. As described above, unlike present-day reef corals, Carnian communities were predominantly composed of corals with solitary and phaceloid growth forms. The Alpe di Specie patch reefs probably developed in relatively muddy, low-energy and photic (or mesophotic) conditions of the back reef (e.g., Fürsich & Wendt, 1977; Russo et al., 1991; Tosti et al., 2014; Roden et al., 2020). Such quiet environments were preferable for phaceloid corals due to their fragile structure (Bernecker, Weidlich & Flügel, 1999; see Martindale, Bottjer & Corsetti, 2012) and mud-sticking adaptations to a muddy substrate (*sensu* Seilacher, 1984; Roniewicz & Stolarski, 1999; Flügel, 2002). Since polyps occupied only the tips of corallites, phaceloid forms were also able to sustain increased sedimentation (Lathuilière et al., 2005).

## Conclusions

Our findings match the scenario that symbiosis preceded the Late Triassic reef bloom. We suggest that the coral-algae relationship was common among corals living on the patch reefs developed in the present-day Alpe di Specie area. The skeletal evidence of symbiosis was found in taxa that previously were considered as azooxanthellate e.g., *Craspedophyllia* and *Margarophyllia* (Kiessling & Kocsis, 2015). This observation suggests that either previous classification of *Craspedophyllia* and *Margarophyllia* as asymbiotic was incorrect (new systematic studies of these taxa, possibly from different locations, are needed) or that both corals, similarly to modern *Tubastrea,* represent ”‘exceptions” from the microstructural criterion. Such possible exceptions from the microstructural criterion call for more in-depth observations of microecology of modern shallow-water corals with mixotrophic nutrition mode. It may appear that in some cases behavioral and/or environmentally controlled factors such as cyclic food supply may influence regularity of biomineralization cycles.

No compelling evidence of asymbiotic species was found among studied Late Triassic coral assemblage. The fossil record of asymbiotic scleractinian corals has long been suggested (macromorphological reconstructions excluded; Stanley & Cairns, 1988; (Gruszczyński et al., 1990; Stanley Jr & Swart, 1995; Gill, Santantonio & Lathuilière, 2004) but only recently, Tornabene et al. (2017) presented reliable geochemical data pointing on azooxanthellate status of Early Miocene *Caryophyllia* sp. Such a result, however, could be expected considering that all shallow- and deep-water extant species of *Caryophyllia* lack photosymbionts. In agreement with previous reports of Late Triassic symbiosis, our results suggest that either all corals were symbiotic or that asymbiotic scleractinians inhabited different niches than shallow-water tropics. Data presented in this study support the conclusion that photosymbiotic corals prevailed in the Early Mesozoic oceans and their partners involved most likely entirely extinct lineages of Symbiodiniaceae (LaJeunesse et al., 2018).

##  Supplemental Information

10.7717/peerj.11062/supp-1 Supplemental Information 1 Inventory numbers of sections, taxonomic attribution, and growth forms of examined Carnian corals from Alpe di Specie

10.7717/peerj.11062/supp-2 Supplemental Information 2 Inventory numbers of sections, taxonomic attribution, and carbon and oxygen isotopic signatures of examined Carnian corals from Alpe di Specie and corresponding calcite infilling of the corallites (the same inventory number as coral sample but with “˙C” e

10.7717/peerj.11062/supp-3 Supplemental Information 3 Inventory numbers of sections, taxonomic attribution, and carbon and oxygen isotopic signatures of examined Carnian corals from Alpe di Specie and corresponding calcite infilling of the corallites (the same inventory number as coral sample but with “_C” e

10.7717/peerj.11062/supp-4 Supplemental Information 4 Presence of growth increments in modern and fossil corals Transmitted light image of (A) modern symbiotic coral *Montipora* sp. (ZPAL H.25/113) and (B) Carnian gen.n. (ZPAL.H.29/18). Note that growth increments in both corals may appear only in some parts of the skeleton, while they are not visible in other areas. Scale bar 100 μ m.

10.7717/peerj.11062/supp-5 Supplemental Information 5 State of preservation of Carnian scleractinian corals used for geochemical analyses Transmitted light images and cathodoluminescence images of: (A, D) volzeiid** sp. A ZPAL H.29/1; (B, E) protoheterastraeid** ZPAL H.29/5; (C, F) *Cuifia* sp. ZPAL H.29/6; (G, J) *Craspedophyllia* sp. ZPAL H.29/8; (H, K) gen.n. C ZPAL H.29/21; (I, L) *Kompsasteria seniora* ZPAL H.29/19. Lack of luminescence in TDs indicates their aragonite mineralogy (D-F, J-L), whereas red luminescence in RADs indicates recrystallization to calcite (D,F,J,K). Sparry calcite cement with red luminescence fills all presented here corallites (D-F, J-L). Scale bars 500 *μ*m.

10.7717/peerj.11062/supp-6 Supplemental Information 6 State of preservation of Carnian scleractinian corals used for geochemical analyses Transmitted light images and cathodoluminescence images of: (A, D) *Margarophyllia capitata* ZPAL H.29/10; (B, E) *M. capitata* ZPAL H.29/11; (C, F) *Margarosmilia communis* ZPAL H.29/15; (G, J) *Margarosmilia montlivatioides* ZPAL H.29/14; (H, K) *Margarastraea klipsteini* ZPAL H.29/16; (I, L) *M. klipsteini* ZPAL H.29/17. Black luminescence in TDs indicates their aragonite composition (D–F, J–L), whereas zones of rapid accretion (RADs) with red luminescence are recrystallized to calcite (D,K). Calcite cement (red color in CL) fills all presented here corallites (D–F, J–L). Scale bars 500 *μ*m.

10.7717/peerj.11062/supp-7 Supplemental Information 7 State of preservation of Carnian scleractinian corals used for geochemical analyses Transmitted light images and cathodoluminescence images of: (A, D) *Tropiastraea carinata* (morphotype A)** ZPAL H.29/30; (B, E) *Tropiastraea carinata* (morphotype C) ZPAL H.29/31; (C, F) tropiastraeiid** sp. B ZPAL H.29/26; (G, J) *Thamnasteriomorpha frechi* ZPAL H.29/34; (H, K) *Thamnasteriomorpha loretzi* ZPAL H.29/33; (I, L) *Thamnasteriomorpha* sp. ZPAL H.29/36. Fibrous parts of the skeleton characterized by black color in CL are composed of aragonite (D–F, J–L), whereas red-luminescent RADs are recrystallized to calcite (D,F). In all cases corallites are filled with sparry calcite cement (red luminescence) (D–F, J–L). Scale bars 500 *μ*m.

10.7717/peerj.11062/supp-8 Supplemental Information 8 State of preservation of Carnian scleractinian corals used for geochemical analyses Transmitted light images and cathodoluminescence images of: (A, D) pamiroseriid ZPAL H.29/22; (B, E) cuifastreiid ZPAL H.29/24; (C, F) conophylliid ZPALH.23/9. Presented skeletons are composed of non-luminescent aragonite (D-F). Presence of non-luminescent aragonite juxtaposed by few-micrometers in size red areas in** pamiroseriid skeleton indicates partial recrystallization of TDs fibers (D). Sparry calcite (red color in CL) occurs in corallite infilling (D-F). Scale bars 500 *μ*m.

10.7717/peerj.11062/supp-9 Supplemental Information 9 Fossil scleractinians from Alpe di Specie showing coefficient of variation (CV) with 95% confidence intervals Specimens with low morphological integration (solitary or phaceloid): (1) volzeiid sp.** B (2) tropiastraeiid** sp. B (3) *Remismilia* sp.** (4) *Craspedophyllia* sp. (5) *Remismilia* sp.** (6) *Margarosmilia* cf. *confluens* (7) conophylliid (8) *Margarosmilia* cf. *confluens* (9) *Margarosmilia montlivatioides* (10) gen. n. B (11) coryphylliid (12) *Retiophyllia* sp. (13) tropiastraeiid** sp. C (14) gen. n. A; specimens with medium morphological integration (cerioid): (15) tropiastraeiid** sp. E (16) *Tropiastraea* sp. (17) tropiastraeiid sp. A (18) tropiastraeiid sp. D (19) and specimens with high morphological integration (thamnasterioid) *Astraeomorpha pratzi* (20) *Thamnasteriomorpha* sp. and (21) *Thamnasteriomorpha frechi*. Green field indicates CV values characteristic for modern symbiotic corals whereas, the yellow field corresponds to CV values of modern asymbiotic corals (based on (Frankowiak et al., 2016a); CC BY NC)

10.7717/peerj.11062/supp-10 Supplemental Information 10 Carbon ( *δ*^13^C) and oxygen ( *δ*^180^) isotopic composition of Carnian corals from Alpe di Specie and corresponding calcite cement from corallites infilling Note the difference between values obtained from skeletons (color-filled symbols) and those of calcite infilling (color empty symbols), paired measurements linked with dashed lines (data in Tab. S3). Areas marked with black line correspond to previous isotopic data from Stanley Jr & Swart (1995) and Frankowiak et al. (2016a); MZ –modern zooxanthellates, MA –modern azooxanthellates, CZ –Carnian zooxanthellates and NZ –Norian zooxanthellate corals.

## Additional Information and Declarations

### Data Availability

The following information was supplied regarding data availability: Raw data is available in the Supplementary Files.

### New Species Registration

The following information was supplied regarding the registration of a newly described species: Formally we are not describing any new taxa in this paper, however we indicate a separate (new) status of some of the taxa using an open nomenclature. A large monograph that will formalize taxonomic status of these corals is in preparation.
