# Supplementary material for: Photosymbiosis in Late Triassic scleractinian corals from the Italian Dolomites
Source: PeerJ. 2021 Mar 16;9:e11062. doi: 10.7717/peerj.11062 (PMC7977380; doi:10.7717/peerj.11062)
Supplement: Supplemental Information 1 [file peerj-09-11062-s001.doc]

SOM Table 1. **Inventory numbers of sections, taxonomic attribution, and growth forms of examined Carnian corals from Alpe di Specie.**

| **Inventory number ZPAL** | **Taxonomic attribution** | **Traditional family** | **Growth form** |
| --- | --- | --- | --- |
| ZPAL.H.29/1 | volzeiid sp. A | Volzeiidae | solitary |
| ZPAL.H.29/2 | volzeiid sp. B | Volzeiidae | solitary |
| ZPAL.H.29/3 | *Remismilia* sp. | Protoheterastreidae | phaceloid |
| ZPAL.H.29/4 | *Remismilia* sp. | Protoheterastreidae | phaceloid |
| ZPAL.H.29/5 | protoheterastraeid | Protoheterastreidae | solitary |
| ZPAL.H.29/6 | *Cuifia* sp. | Coryphylliidae | solitary |
| ZPAL.H.29/7 | coryphylliid | Coryphylliidae | phaceloid |
| ZPAL.H.29/8 | *Craspedophyllia* sp. | Reimaniphyllidae | solitary |
| ZPAL.H.29/9 | *Retiophyllia* sp. | Reimaniphyllidae | phaceloid |
| ZPAL.H.29/10 | *Margarophyllia capitata* | Margarophylliidae | solitary |
| ZPAL.H.29/11 | *Margarophyllia capitata* | Margarophylliidae | solitary |
| ZPAL.H.29/12 | *Margarosmilia* cf. *confluens* | Margarophylliidae | phaceloid |
| ZPAL.H.29/13 | *Margarosmilia* cf. *confluens* | Margarophylliidae | phaceloid |
| ZPAL.H.29/14 | *Margarosmilia montlivatioides* | Margarophylliidae | phaceloid |
| ZPAL.H.29/15 | *Margarosmilia communis* | Margarophylliidae | phaceloid |
| ZPAL.H.29/16 | *Margarastraea klipsteini* | Margarophylliidae | meandroid |
| ZPAL.H.29/17 | *Margarastraea klipsteini* | Margarophylliidae | meandroid |
| ZPAL.H.29/18 | gen. n. A | fam. n. | solitary |
| ZPAL.H.29/19 | *Kompsasteria seniora* | Cycliphylliidae | cerioid |
| ZPAL.H.29/20 | gen. n. B | fam. n. | solitary |
| ZPAL.H.29/21 | gen. n. C | fam. n. | cerioid |
| ZPAL.H.29/22 | pamiroseriid | Pamiroseriidae | cerioid |
| ZPAL.H.29/23 | cuifastreiid | Cuifastraeidae | solitary |
| ZPAL.H.29/24 | cuifastreiid | Cuifastraeidae | solitary |
| ZPAL.H.29/25 | tropiastraeiid sp. A | Tropiastraeidae | cerioid |
| ZPAL.H.29/26 | tropiastraeiid sp. B | Tropiastraeidae | solitary |
| ZPAL.H.29/27 | tropiastraeiid sp. C | Tropiastraeidae | solitary |
| ZPAL.H.29/28 | tropiastraeiid sp. D | Tropiastraeidae | cerioid |
| ZPAL.H.29/29 | tropiastraeiid sp. E | Tropiastraeidae | cerioid |
| ZPAL.H.29/30 | *Tropiastraea carinata* A | Tropiastraeidae | cerioid |
| ZPAL.H.29/31 | *Tropiastraea carinata* C | Tropiastraeidae | cerioid |
| ZPAL.H.29/32 | *Tropiastraea* sp. | Tropiastraeidae | cerioid |
| ZPAL.H.29/33 | *Thamnasteriamorpha loretzi* | Tropiastraeidae | thamnasterioid |
| ZPAL.H.29/34 | *Thamnasteriamorpha frechi* | Tropiastraeidae | thamnasterioid |
| ZPAL.H.29/35 | *Thamnasteriamorpha frechi* | Tropiastraeidae | thamnasterioid |
| ZPAL.H.29/36 | *Thamnasteriamorpha* sp. | Tropiastraeidae | thamnasterioid |
| ZPAL.H.29/37 | *Astraeomorpha pratzi* | Astraeomorphidae | thamnasterioid |
| ZPALH.23/9 | conophylliid | Conophyllidae | solitary |
