# Supplementary material for: Photosymbiosis in Late Triassic scleractinian corals from the Italian Dolomites
Source: PeerJ. 2021 Mar 16;9:e11062. doi: 10.7717/peerj.11062 (PMC7977380; doi:10.7717/peerj.11062)
Supplement: Supplemental Information 2 [file peerj-09-11062-s002.doc]

SOM Table 2. **Inventory numbers of sections, taxonomic attribution, and regularity of growth increments [expressed as coefficient of variation (%) of dispersion of values of band thickness obtained from each skeleton] of examined Carnian corals from Alpe di Specie.**

| **Inventory number ZPAL** | **Number in Supplemental Figure S6** | **Taxonomic attribution** | **Coefficient of variation [%]** |
| --- | --- | --- | --- |
| ZPAL.H.29/2 | 1 | volzeiid sp. B | 7 |
| ZPAL.H.29/3 | 3 | *Remismilia* sp. | 5 |
| ZPAL.H.29/4 | 5 | *Remismilia* sp. | 9 |
| ZPAL.H.29/7 | 11 | coryphylliid | 15 |
| ZPAL.H.29/8 | 4 | *Craspedophyllia* sp. | 13 |
| ZPAL.H.29/9 | 12 | *Retiphyllia* sp. | 7 |
| ZPAL.H.29/12 | 6 | *Margarosmilia* cf. *confluens* | 8 |
| ZPAL.H.29/13 | 8 | *Margarosmilia* cf. *confluens* | 10 |
| ZPAL.H.29/14 | 9 | *Margarosmilia montlivatioides* | 6 |
| ZPAL.H.29/18 | 14 | gen. n. A | 8 |
| ZPAL.H.29/20 | 10 | gen. n. B | 7 |
| ZPAL.H.29/25 | 17 | tropiastraeiid sp. A | 6 |
| ZPAL.H.29/26 | 2 | tropiastraeiid sp. B | 9 |
| ZPAL.H.29/27 | 13 | tropiastraeiid sp. C | 12 |
| ZPAL.H.29/28 | 18 | tropiastraeiid sp. D | 4 |
| ZPAL.H.29/29 | 15 | tropiastraeiid sp. E | 6 |
| ZPAL.H.29/32 | 16 | *Tropiastraea* sp. | 9 |
| ZPAL.H.29/35 | 21 | *Thamnasteriomorpha frechi* | 40 |
| ZPAL.H.29/36 | 20 | *Thamnasteriomorpha* sp. | 41 |
| ZPAL.H.29/37 | 19 | *Astraeomorpha pratzi* | 9 |
| ZPALH.23/9 | 7 | conophylliid | 6 |
