# Supplementary material for: Photosymbiosis in Late Triassic scleractinian corals from the Italian Dolomites
Source: PeerJ. 2021 Mar 16;9:e11062. doi: 10.7717/peerj.11062 (PMC7977380; doi:10.7717/peerj.11062)
Supplement: Supplemental Information 3 [file peerj-09-11062-s003.doc]

SOM Table 3. **Inventory numbers of sections, taxonomic attribution, and carbon and oxygen isotopic signatures of examined Carnian corals from Alpe di Specie and corresponding calcite infilling of the corallites (the same inventory number as coral sample but with “_C” ending) (AS-aragonite skeleton; CS-calcite skeleton).**

| **Inventory number ZPAL** | **Taxonomic attribution** | **δ^13^C [‰]** | **δ^18^O [‰]** | **Inventory number** | **δ^13^C [‰]** | **δ^18^O [‰]** |
| --- | --- | --- | --- | --- | --- | --- |
| ZPAL.H.29/1 | volzeiid sp. A | 3.49 | -3.11 | ZPAL.H.29/1_C | 3.38 | -2.95 |
| ZPAL.H.29/5 | protoheterastraeid | 3.19 | -2.74 | ZPAL.H.29/5_C | 2.75 | -1.57 |
| ZPAL.H.29/6 | *Cuifia* sp. | 3.37 | -3.46 | ZPAL.H.29/6_C | -0.31 | -4.03 |
| ZPAL.H.29/8 | *Craspedophyllia* sp. | 4.46 | -3.23 | ZPAL.H.29/8_C | 2.34 | -2.30 |
| ZPAL.H.29/10 | *Margarophyllia capitata* | 3.98 | -2.30 | ZPAL.H.29/10_C | 2.09 | -2.84 |
| ZPAL.H.29/11 | *Margarophyllia capitata* | 3.55 | -1.06 | ZPAL.H.29/11_C | 1.63 | -2.89 |
| ZPAL.H.29/14 | *Margarosmilia montlivatioides* | 3.56 | -2.43 | ZPAL.H.29/14_C | 1.93 | -3.22 |
| ZPAL.H.29/15 | *Margarosmilia communis* | 3.87 | -3.25 | ZPAL.H.29/15_C | 2.31 | -1.83 |
| ZPAL.H.29/16 | *Margarastraea klipsteini* | 2.36 | -3.14 | ZPAL.H.29/16_C | 1.95 | -2.57 |
| ZPAL.H.29/17 | *Margarastraea klipsteini* | 3.19 | -3.77 | ZPAL.H.29/17_C | 1.96 | -1.85 |
| ZPAL.H.29/19 | *Kompsasteria seniora* | 0.81 | -3.35 | ZPAL.H.29/19_C | -3.11 | -3.70 |
| ZPAL.H.29/21 | gen. n. C | 1.99 | -3.88 |  |  |  |
| ZPAL.H.29/22 | pamiroseriid | 4.93 | -3.04 | ZPAL.H.29/22_C | 2.88 | -2.84 |
| ZPAL.H.29/23 | cuifastreiid | 5.81 | -2.74 |  |  |  |
| ZPAL.H.29/24 | cuifastreiid | 4.30 | -1.90 | ZPAL.H.29/24_C | 3.71 | -2.89 |
| ZPAL.H.29/26 | tropiastraeiid sp. B | 3.94 | -2.67 | ZPAL.H.29/26_C | 2.53 | -2.39 |
| ZPAL.H.29/29 | tropiastraeiid sp. E AS | 4.59 | -3.30 |  |  |  |
| ZPAL.H.29/29 | tropiastraeiid sp. E CS | 4.73 | -3.02 |  |  |  |
| ZPAL.H.29/30 | *Tropiastraea carinata* A | 0.93 | -2.98 | ZPAL.H.29/30_C | 0.93 | -5.09 |
| ZPAL.H.29/31 | *Tropiastraea carinata* C | 4.82 | -2.46 | ZPAL.H.29/31_C | 3.31 | -2.36 |
| ZPAL.H.29/33 | *Thamnasteriomorpha loretzi* | 4.25 | -2.51 |  |  |  |
| ZPAL.H.29/34 | *Thamnasteriomorpha frechi* | 3.61 | -2.81 |  |  |  |
| ZPAL.H.29/36 | *Thamnasteriomorpha* sp. | 2.51 | -2.90 |  |  |  |
| ZPALH.23/9 | conophylliid | 3.25 | -4.21 |  |  |  |
|  |  |  |  |  |  |  |
