# Supplementary figures and images for: Photosymbiosis in Late Triassic scleractinian corals from the Italian Dolomites
Source: PeerJ. 2021 Mar 16;9:e11062. doi: 10.7717/peerj.11062 (PMC7977380; doi:10.7717/peerj.11062)

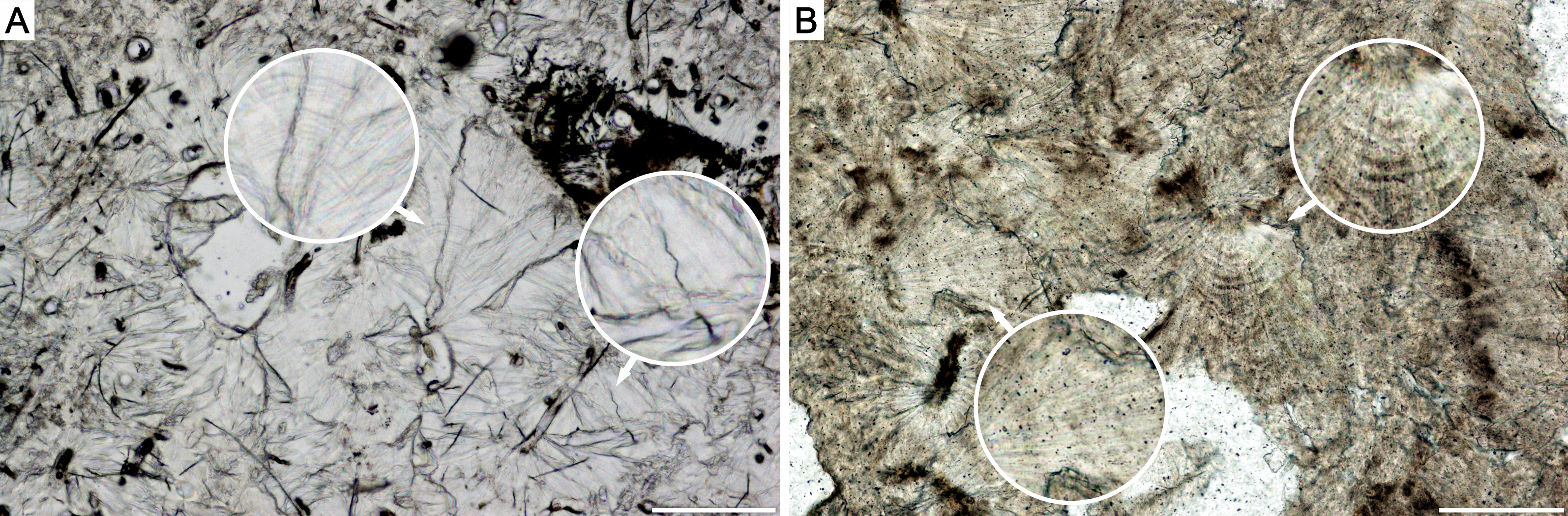

Supplement: Supplemental Information 4 — Transmitted light image of (A) modern symbiotic coral Montipora sp. (ZPAL H.25/113) and (B) Carnian gen.n. (ZPAL.H.29/18). Note that growth increments in both corals may appear only in some parts of the skeleton, while they are not visible in other areas. Scale bar 100 μ m. [file peerj-09-11062-s004.png]

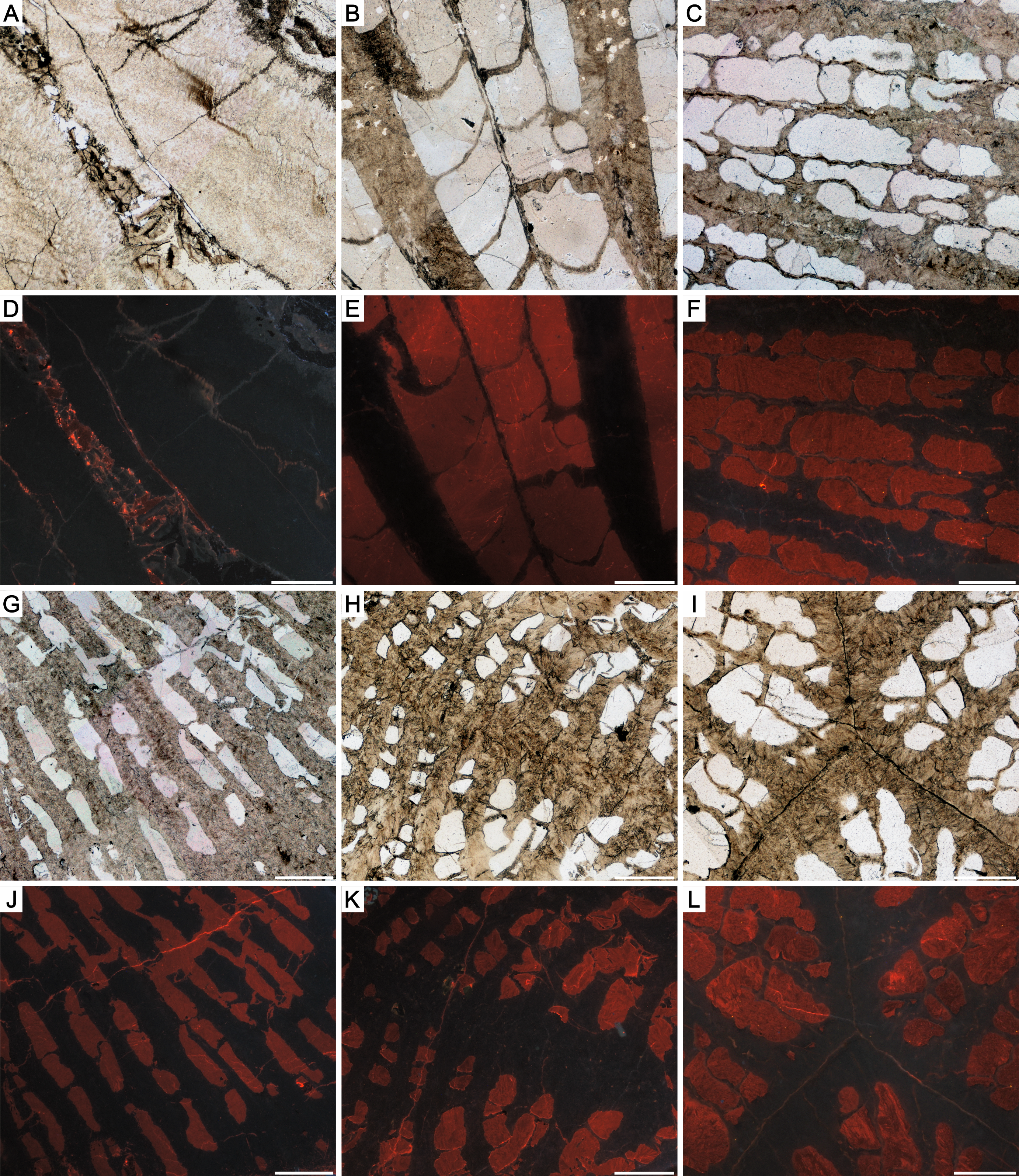

Supplement: Supplemental Information 5 — Transmitted light images and cathodoluminescence images of: (A, D) volzeiid sp. A ZPAL H.29/1; (B, E) protoheterastraeid ZPAL H.29/5; (C, F) Cuifia sp. ZPAL H.29/6; (G, J) Craspedophyllia sp. ZPAL H.29/8; (H, K) gen.n. C ZPAL H.29/21; (I, L) Kompsasteria seniora ZPAL H.29/19. Lack of luminescence in TDs indicates their aragonite mineralogy (D-F, J-L), whereas red luminescence in RADs indicates recrystallization to calcite (D,F,J,K). Sparry calcite cement with red luminescence fills all presented here corallites (D-F, J-L). Scale bars 500 μm. [file peerj-09-11062-s005.png]

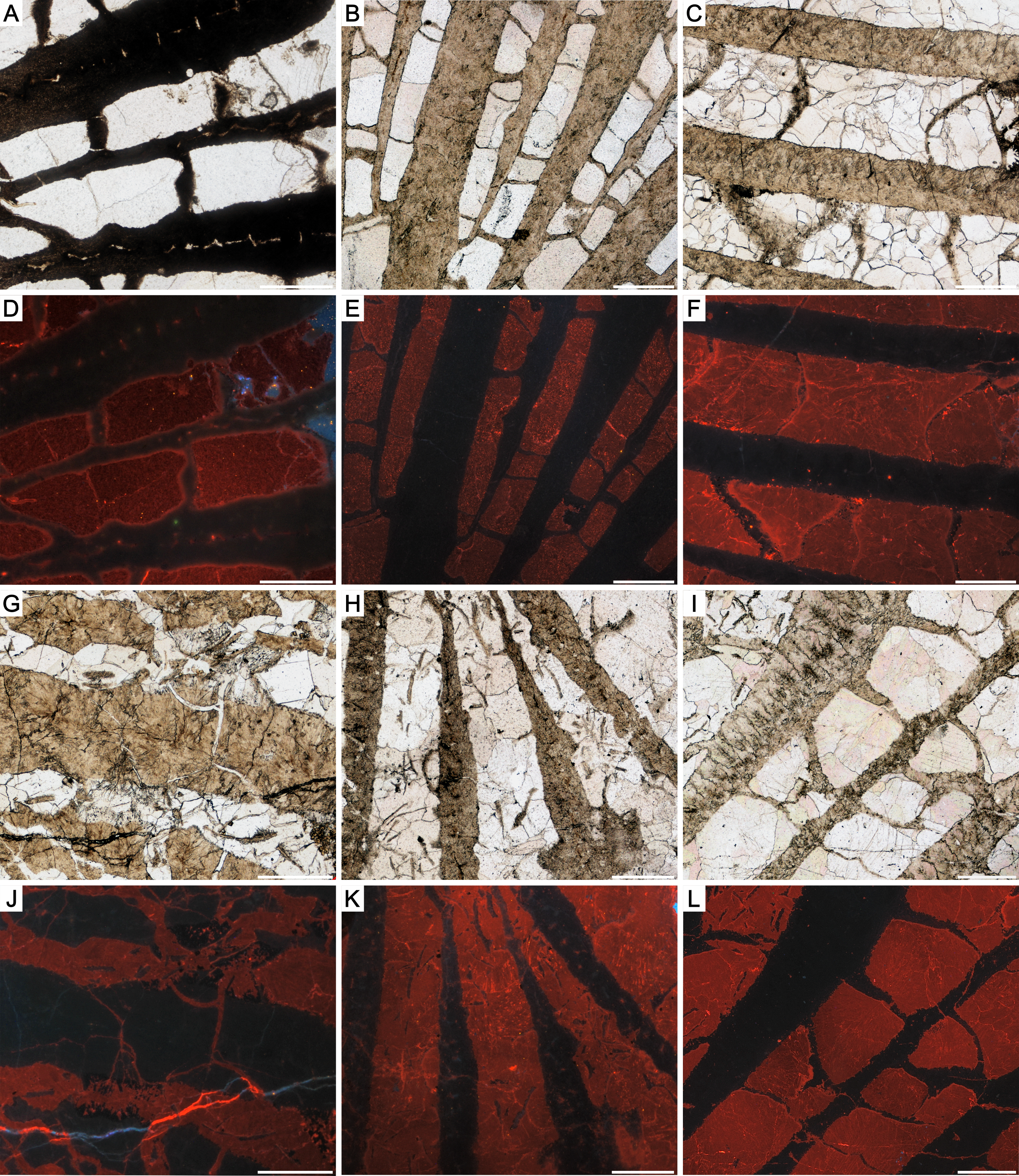

Supplement: Supplemental Information 6 — Transmitted light images and cathodoluminescence images of: (A, D) Margarophyllia capitata ZPAL H.29/10; (B, E) M. capitata ZPAL H.29/11; (C, F) Margarosmilia communis ZPAL H.29/15; (G, J) Margarosmilia montlivatioides ZPAL H.29/14; (H, K) Margarastraea klipsteini ZPAL H.29/16; (I, L) M. klipsteini ZPAL H.29/17. Black luminescence in TDs indicates their aragonite composition (D–F, J–L), whereas zones of rapid accretion (RADs) with red luminescence are recrystallized to calcite (D,K). Calcite cement (red color in CL) fills all presented here corallites (D–F, J–L). Scale bars 500 μm. [file peerj-09-11062-s006.png]

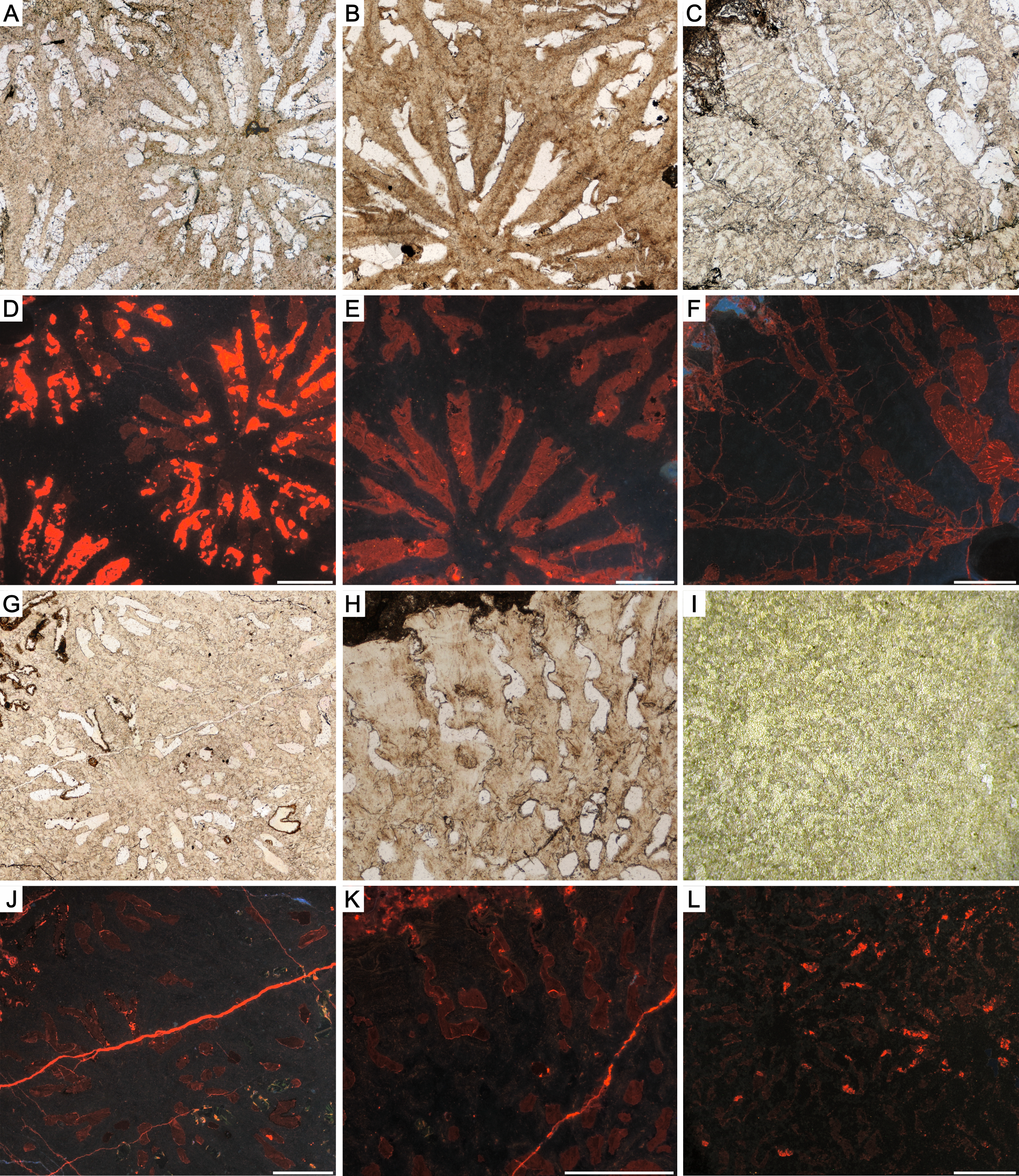

Supplement: Supplemental Information 7 — Transmitted light images and cathodoluminescence images of: (A, D) Tropiastraea carinata (morphotype A) ZPAL H.29/30; (B, E) Tropiastraea carinata (morphotype C) ZPAL H.29/31; (C, F) tropiastraeiid sp. B ZPAL H.29/26; (G, J) Thamnasteriomorpha frechi ZPAL H.29/34; (H, K) Thamnasteriomorpha loretzi ZPAL H.29/33; (I, L) Thamnasteriomorpha sp. ZPAL H.29/36. Fibrous parts of the skeleton characterized by black color in CL are composed of aragonite (D–F, J–L), whereas red-luminescent RADs are recrystallized to calcite (D,F). In all cases corallites are filled with sparry calcite cement (red luminescence) (D–F, J–L). Scale bars 500 μm. [file peerj-09-11062-s007.png]

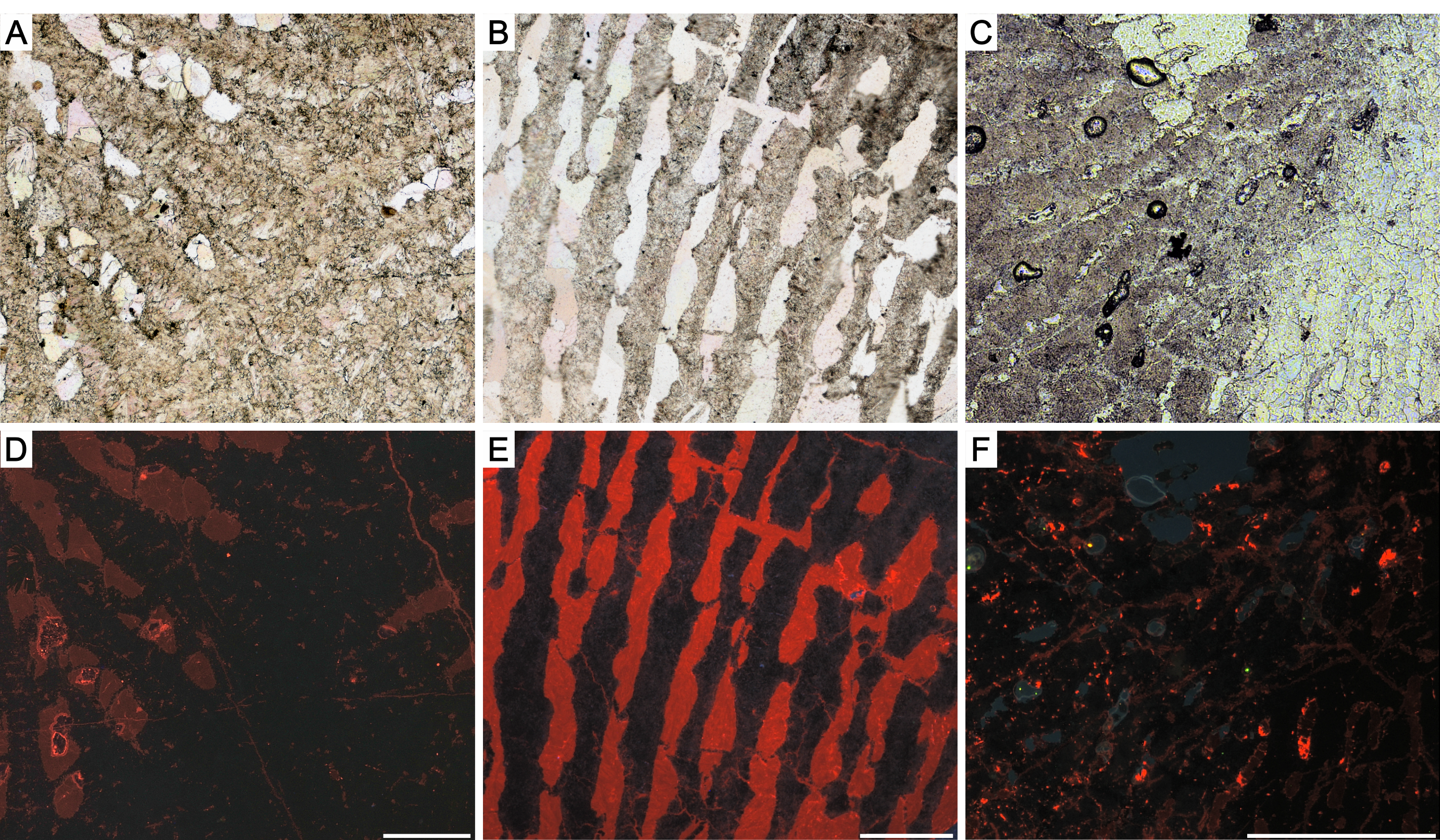

Supplement: Supplemental Information 8 — Transmitted light images and cathodoluminescence images of: (A, D) pamiroseriid ZPAL H.29/22; (B, E) cuifastreiid ZPAL H.29/24; (C, F) conophylliid ZPALH.23/9. Presented skeletons are composed of non-luminescent aragonite (D-F). Presence of non-luminescent aragonite juxtaposed by few-micrometers in size red areas in pamiroseriid skeleton indicates partial recrystallization of TDs fibers (D). Sparry calcite (red color in CL) occurs in corallite infilling (D-F). Scale bars 500 μm. [file peerj-09-11062-s008.png]

# morphological integration

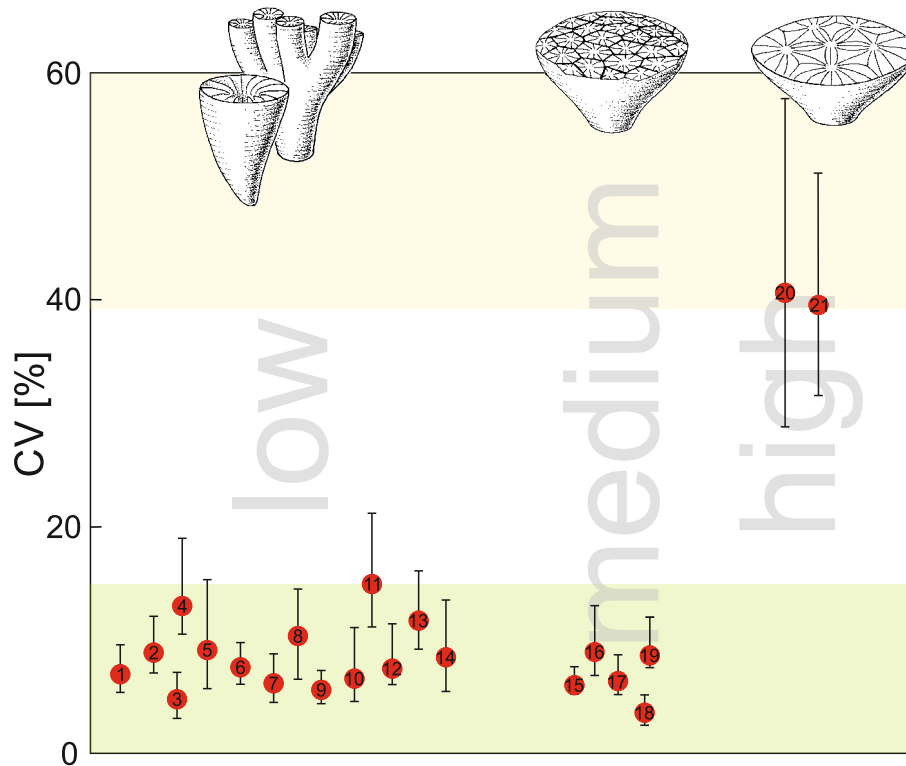

Supplement: Supplemental Information 9 — Specimens with low morphological integration (solitary or phaceloid): (1) volzeiid sp. B (2) tropiastraeiid sp. B (3) Remismilia sp. (4) Craspedophyllia sp. (5) Remismilia sp. (6) Margarosmilia cf. confluens (7) conophylliid (8) Margarosmilia cf. confluens (9) Margarosmilia montlivatioides (10) gen. n. B (11) coryphylliid (12) Retiophyllia sp. (13) tropiastraeiid sp. C (14) gen. n. A; specimens with medium morphological integration (cerioid): (15) tropiastraeiid sp. E (16) Tropiastraea sp. (17) tropiastraeiid sp. A (18) tropiastraeiid sp. D (19) and specimens with high morphological integration (thamnasterioid) Astraeomorpha pratzi (20) Thamnasteriomorpha sp. and (21) Thamnasteriomorpha frechi. Green field indicates CV values characteristic for modern symbiotic corals whereas, the yellow field corresponds to CV values of modern asymbiotic corals (based on (Frankowiak et al., 2016a); CC BY NC) [file peerj-09-11062-s009.pdf]

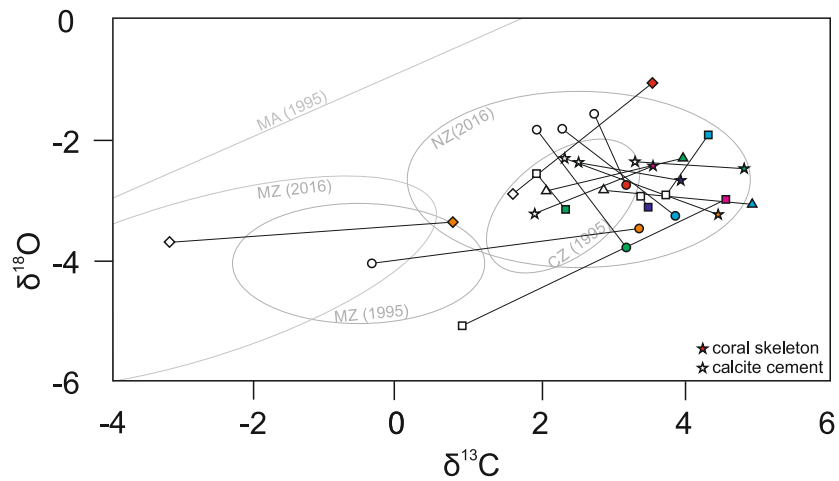

Supplement: Supplemental Information 10 — Note the difference between values obtained from skeletons (color-filled symbols) and those of calcite infilling (color empty symbols), paired measurements linked with dashed lines (data in Tab. S3). Areas marked with black line correspond to previous isotopic data from Stanley Jr & Swart (1995) and Frankowiak et al. (2016a); MZ –modern zooxanthellates, MA –modern azooxanthellates, CZ –Carnian zooxanthellates and NZ –Norian zooxanthellate corals. [file peerj-09-11062-s010.pdf]
